# High quality, small molecule-activity datasets for kinase research

**DOI:** 10.12688/f1000research.8950.3

**Published:** 2016-10-26

**Authors:** Rajan Sharma, Stephan C. Schürer, Steven M. Muskal

**Affiliations:** 1 Eidogen-Sertanty, Inc., Oceanside, CA, 92056, USA; 2 Department of Pharmacology, Miller School of Medicine and Center for Computational Science, University of Miami, Miami, FL, 33136, USA

**Keywords:** Kinase, SAR, Bioactivity Database, Dataset, Drug Discovery, Bioactive Molecules, Kinase Knowledgebase, KKB

## Abstract

Kinases regulate cell growth, movement, and death. Deregulated kinase activity is a frequent cause of disease. The therapeutic potential of kinase inhibitors has led to large amounts of published structure activity relationship (SAR) data. Bioactivity databases such as the Kinase Knowledgebase (KKB), WOMBAT, GOSTAR, and ChEMBL provide researchers with quantitative data characterizing the activity of compounds across many biological assays. The KKB, for example, contains over 1.8M kinase structure-activity data points reported in peer-reviewed journals and patents. In the spirit of fostering methods development and validation worldwide, we have extracted and have made available from the KKB 258K structure activity data points and 76K associated unique chemical structures across eight kinase targets. These data are freely available for download within this data note.

## Introduction

Since their discovery in 1975 by Cohen *et al*. ^1^, kinases are now one of the most established drug target families, second only to G-protein-coupled receptors (GPCRs). Most progress in kinase research has occurred in the last 25 years including the discovery of many new kinases ^2, 3^, identification of new isoforms of pre-existing kinases ^4, 5^, elucidation of new biological pathways, and identification of many new kinase-disease associations ^6, 7^. While kinases are well-validated anti-cancer targets ^8– 11^, kinase inhibitors also have been pursued in cardiovascular ^12^, autoimmune ^13^, inflammatory skin and bowel ^14^, neurodegenerative ^15^, and renal disease programs ^16^. Most small-molecule kinase inhibitors target the ATP binding site of the kinase catalytic domain ^11^. The ATP binding region of the catalytic domain is highly conserved among protein kinases, which has important consequences for drug development. Achieving selectivity of a small molecule inhibitor against kinase off-targets to avoid adverse reactions can be a major hurdle. However, the cross reactivity of many chemotypes can also open opportunities to focus on other closely related kinases. Despite the high degree of conservation in the ATP binding site, reasonably selective inhibitors with favorable pharmacological properties can be developed ^17^. It is now common in discovery programs to profile inhibitors against an extensive set of kinase targets ^18^. These kinase-profiling efforts have generated valuable data, providing insight into selectivity and promiscuity of clinical inhibitors ^19– 21^.

Medicinal chemists can benefit significantly from well-curated databases documenting chemical structure(s) with an experimentally measured biological activity. These structure and activity databases or SAR databases help to better understand drug-target interaction, which can assist in the design of potent and selective chemical inhibitors ^22– 25^. A well populated, editable, easy to search and flexible SAR database is an integral part of the modern drug design process ^26^. SAR databases provide elementary insights to researchers, including:

(a) Target druggability: known small molecule binders are required to categorize a protein as druggable. High-affinity and non-promiscuous inhibitors are particularly valuable to establish druggability; and can be further validated using structure biology information. In many cases druggability can be inferred for new targets using homology models ^27^ where similarities can be mapped via sequences, pathways or functions. Examples include the Target Informatics Platform ( TIP) ^28^ and Modbase ^29^.
(b) Scaffold selectivity: the golden principle that applies is “less selective scaffolds have more undesirable side effects.” A prior knowledge of selectivity profiles can help in making informed decisions on which chemotypes to pursue at the start of discovery programs ^30^. Organizing data by scaffold enables classic SAR analysis in which side-chain moieties can be evaluated and considered or avoided in lead optimization ^31^.
(c) Clinical molecules: it can be very helpful to see scaffold(s) or derivatives under the study of launched drugs. This enables medicinal chemists to associate therapeutic classes with active scaffolds.
(d) Development and validation of computational methods: well-curated datasets are very helpful in the development and refinement of computational methodologies. With a common set of data, computational researchers can also compare and contrast methods, providing additional validation ^32^.
(e) Virtual screening: high-quality, well-curated, standardized and annotated datasets are required to build predictive models for virtual screening as we have shown previously specifically for the Kinase Knowledgebase ( KKB) data ^33^.

## Materials and Methods

The KKB is a database of biological activity data, structure-activity relationships, and chemical synthesis data focused on protein kinases. Since its inception in 2001, the KKB has grown steadily with quarterly updates each year. With more than two decades of high quality SAR data, the KKB represents one of the first kinase target specific databases of biological activity and chemical synthesis data from curated scientific literature and patents. The KKB contains a large number of kinase structure-activity data points (>1.8M) reported in peer-reviewed literature covering journals and patents. The data have been curated from over 150 different journals reporting kinase inhibitors with activity data, with leading contributions from *J Med Chem*, *Bioorg Med Chem*, *Bioorg Med ChemLett* and *Euro J Med Chem*. In addition, the KKB contains data curated from patents/applications from WO, EP and US. The scientific information is curated from the published text using a combination of automatic and manual efforts.

A summary of the first quarter release for year 2016 (Q1-2016) is reported in Table 1. With the Q1-2016 KKB release, there are total of 506 unique kinase targets with over 682K unique small molecules. A listing of few “hot” kinase targets with their inhibitors (data points) is reported in Table 2.

**Table 1.** Eidogen-Sertanty Kinase Knowledgebase. Summary Statistics – Q1 2016 Release.

Articles covered:	2,780
Patents and patent applications covered:	6,346
Total Number of Bio-activity data points:	1,775,368
Total Number of unique molecules:	682,289
Total Number of unique molecules w/ assay data:	337,491
Total Number of assay protocols:	32,462

**Table 2.** Eidogen-Sertanty Kinase Knowledgebase. Data Points for Selected Targets– Q1 2016 Release.

Kinase Classification			Enzyme Assay			Cell-Based Assay		
	Family	Target Name	All SAR Data Points	All IC50 Data Points	Unique Assay Molecules	All SAR Data Points	All IC50 Data Points	Unique Assay Molecules
**Non-Receptor** **Tyrosine Kinases**	Abl	**ABL1**	14750	4843	2177	4237	1836	1098
	Csk	**CSK**	3792	1448	450	548	266	146
	Fak	**FAK/PTK2**	10311	4067	3863	2880	1306	1300
	JakA	**JAK3**	29550	8778	11456	1327	605	440
	Src	**SRC**	21936	8289	4480	3425	1473	747
		**LCK**	23819	10514	6090	784	381	214
		**FYN**	3125	873	151	28	11	7
	Syk	**SYK**	39426	17549	16774	1037	484	268
		**ZAP70**	5951	2998	1013	5	2	2
	Tec	**ITK**	10131	3690	2197	219	83	113
**Receptor** **Tyrosine Kinases**	EGFR	**EGFR**	34293	14684	6593	19731	9068	3321
		**ERBB2**	11182	5199	1756	7988	4115	1803
	Eph	**EPHA2**	2935	765	223	12	0	1
	FGFR	**FGFR1**	19582	8394	4149	8781	3345	1622
	InsR	**INSR**	4607	1293	1032	920	422	395
	Met	**MET**	27032	10406	9308	5147	2526	1983
	PDGFR	**PDGFRB**	14058	5889	2388	5426	2653	983
		**FLT3/FLK2**	13082	3974	2830	10224	4386	2268
		**KIT**	14991	5153	2527	7040	3339	2747
	Tie	**TEK**	9142	4306	2300	3122	1561	1360
	Trk	**NTRK1/TRKA**	8199	3207	2925	1743	814	563
	VEGFR	**KDR/FLK1**	55991	24821	13899	20317	9119	6541
		**FLT1**	9963	4251	1116	864	432	197
**CMGC Kinases**	CDK	**CDK2**	33878	12695	10411	5344	1119	667
		**CDK5**	8227	3048	1714	18	3	3
	GSK	**GSK3B**	22950	7766	6992	2013	519	832
	MAPK	**MAPK14**	36067	16077	14270	6541	2373	2787
		**MAPK1**	11286	3073	3081	2725	1064	1085
		**MAPK10**	5725	1615	1610	96	48	23
		**MAPK8**	6225	1803	1523	880	285	393
		**MAPK11**	1162	196	100	0	0	0
**AGC Kinases**	AKT	**AKT1**	14601	6333	5794	6970	3064	2831
	DMPK	**ROCK1**	9135	2052	3105	189	40	65
	PKB	**PDPK1**	9569	3765	2642	148	68	44
	PKC	**PRKCA**	10670	3528	2588	5477	669	510
		**PRKCE**	3759	1494	1032	2	1	1
**CAMK Kinases**	CAMKL	**CHEK1**	13724	5192	5202	3140	220	1130
	MAPKAPK	**MAPKAPK2**	11041	4073	3747	1311	649	637
		**MAPKAPK3**	2138	518	299	0	0	0
**Other Protein** **Kinases**	AUR	**AURKA**	22646	7904	7034	1128	474	382
	IKK	**IKBKB**	7628	2978	3146	367	83	144
		**CHUK/IKBKA**	2938	999	764	296	148	147
	PLK	**PLK1**	9181	3223	3480	2986	1364	888
	STE	**MAP2K1**	6340	2551	2045	1651	573	655
	TKL	**ILK**	360	180	172	581	253	80
		**RAF1**	11302	5058	3378	1956	885	581
		**BRAF**	26349	12169	8983	6726	2442	2106
**Other Non-** **Protein Kinases**	Lipid Kinases	**PIK3/PIK3CG**	29925	13438	10899	3525	1758	1217
		**PIK3CA**	36168	16418	12448	3392	1310	1219
	Nucleotide Kinases	**TK1**	1106	301	339	2416	533	193
		**ADK**	1924	931	723	669	252	240

Kinase inhibitors are biologically active small molecules and their activity refers to experimentally measured data on a given kinase target (in enzyme or in cell based assays), using predefined experimental protocols. After curation and standardization, these measured values together with related information are indexed in the KKB. Each inhibitor entered in the KKB carries unique identifiers such as:

(a) Chemical information and biological information: unique structure IDs (MR_ID) are assigned based on unique canonical SMILES. In addition hand-drawn Cartesian coordinates are captured. Chemical compounds are associated with calculated chemical and physical properties.
(b) Biological target and assay protocol: biological targets are annotated by EntrezGeneID, UniProt ID, and HUGO approved names. An assay protocol includes detailed information pertaining to the experiments performed to measure the biological activity for the compound. Each protocol has a descriptive title and a unique set of keywords. Assays are categorized by assay format (biochemical, cell-based, etc.) following standards set forth by BioAssay Ontology (BAO) ^34, 35^. Kinase targets are classified by protein and non-protein kinases and protein kinases by the typical domain-based classification into group, family, etc. We are in the process of mapping KKB targets to the Drug Target Ontology ( DTO), which is in development.
(c) Experimental bioactivity screening results. A bioactivity data point is a defined result/endpoint of a specified small molecule compound tested in a biological assay. The assay is defined in b); result type/endpoint captured include IC _50_, K _i_, K _d_; the vast majority for biochemical and cell-based assays correspond to BAO definitions.
(d) Source reference: bibliographic information and unique identifiers for journal article and patents from which information related to the molecules was extracted include PubMedID, DOI, and standardized patent numbers. For journals, the KKB provides title, authors name, journal-name, volume, issues, and page numbers. For patents their titles, patent or patent application number (along with family members), inventor’s names, assignee names, publication data and priority numbers are provided.

It is observed that a disease type can be related to multiple kinase groups, and several diseases can arise from a common set of kinase group ( Table 3) ^6^. In the KKB, kinases are classified by protein and non-protein kinases with several sub-categories such as carbohydrate and lipid kinase and the typical protein kinase groups (such CMGC, CAMK, TK, TKL, RGC, AGC) and further sub-groups such as families. DTO provides a functional and phylogenetic classification of kinase domains to facilitate navigation of kinase drug targets. DTO is developed as part of the Illuminating the Druggable Genome (IDG) project. Here we make datasets freely available for the research community including to support efforts such as IDG. We also offer to run our predictive models built using KKB data to support prioritization of drug targets.

**Table 3.** Kinase-disease association in top therapeutic segments.

Disease Class	Kinase Group
Cancer	AGC;atypical;CAMK;CK1; CMGC;RGC;STE;TK;TKL
Diabetes	AGC;CMGC;TK
Cardiovascular	AGC;CAMK;CMGC;TKL
Hypertension	AGC;CAMK;RGC
Neurodegeneration	AGC;CAMK;CMGC;CK1
Inflammation	CMGC;STE;TKL
Immunity	AGC;TK

## Kinase inhibitor datasets

The wealth of kinase inhibitor data presents opportunities for analysis as a whole or by integrating such data into various computational platforms to support development and validation of hypotheses of kinase inhibition. Several years ago, Eidogen-Sertanty made available 3880 pIC _50_ data points across three kinase targets (ABL1, SRC, and AURKA – validation sets) to foster algorithm development and validation worldwide. With this data note, eight additional targets comprising inhibitors for therapeutically important classes: EGFR, CDK2, ROCK2, MAPK14 and PI3K (class I catalytic) ( Table 4) totaling ~258K data points (structure with standard results/endpoints such as IC _50_, K _i_ or K _d_) and ~76K unique chemical structures now have been made available to further foster worldwide development, validation, and collaborative interaction (see KB_SAR_DATA_F1000.txt and KB_SAR_DATA_F1000.sdf files). These datapoints have been exported from the KKB and survey 1044 articles and 942 patents.

**Table 4.** Important aspects about the selected targets.

Kinase	Approved Name	Class	Diseases Associated	Entrez GeneID	Uniprot ID
EGFR*	Epidermal Growth Factor Receptor	Receptor Tyrosine Kinase	NSCLC, Medullary Thyroid Cancer, Breast Cancer, Neonatal Inflammatory Skin and Bowel Disease	1956	P00533
CDK2	Cyclin-Dependent Kinase 2	Serine/Threonine Kinase	Angiomyoma, Carbuncle	1017	P24941
ROCK2	Rho-Associated, Coiled-Coil Containing Protein Kinase 2	Serine/Threonine Kinase	Colorectal Cancer, Penile Disease, Hepatocellular Carcinoma	9475	O75116
MAPK14	Mitogen-Activated Protein Kinase 14	Serine/Threonine Kinase	Acquired Hyperkeratosis, Prostate Transitional Cell Carcinoma, Immunity-related Diseases	1432	Q16539
PIK3CA	Phosphatidylinositol-4,5- Bisphosphate 3-Kinase, Catalytic Subunit Alpha	Lipid Kinase	Colorectal Cancer, Actinic Keratosis	5290	P42336
PIK3CB	Phosphatidylinositol-4,5- Bisphosphate 3-Kinase, Catalytic Subunit Beta	Lipid Kinase	-	5291	P42338
PIK3CD	Phosphatidylinositol-4,5- Bisphosphate 3-Kinase, Catalytic Subunit Delta	Lipid Kinase	Immunodeficiency 14, Activated PIK3-Delta Syndrome	5293	O00329
PIK3CG	Phosphatidylinositol-4,5- Bisphosphate 3-Kinase, Catalytic Subunit Gamma	Lipid Kinase	Lichen Nitidus	5294	P48736

*Afatinib, Erlotinib, Gefitinib, Lapatinib, Osimertinib, Vandetanib are US-FDA approved kinase inhibitors with EGFR as one of the valid targets.

The datasets cover a broad range of biochemical and cell based studies investigating kinase inhibition; and they represent a diverse collection of pharmaceutically active scaffolds. These scaffolds can be easily examined for selectivity and specificity for the given eight kinase targets. Additionally, they can be used to infer novel target-inhibitor relationships for kinases and compounds not included in these subsets.

Bibliographic information is reported in the files ArticleInfo_F1000.txt and PatentInfo_F1000.txt. Experimental procedure along with metadata information for targets including EntrezGeneIDs, assay format/type (biochemical/enzyme, cell based, etc), keywords, species, and cell lines used in cell-based data are stored in AssayProtocols_F1000 (txt and xml attached).

**Figure 1.**
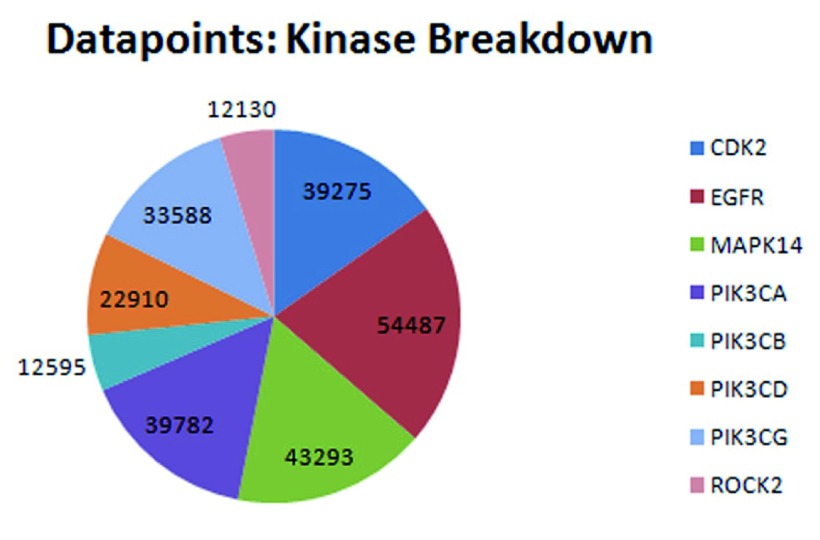
Data point distributions for each kinase.

**Figure 2.**
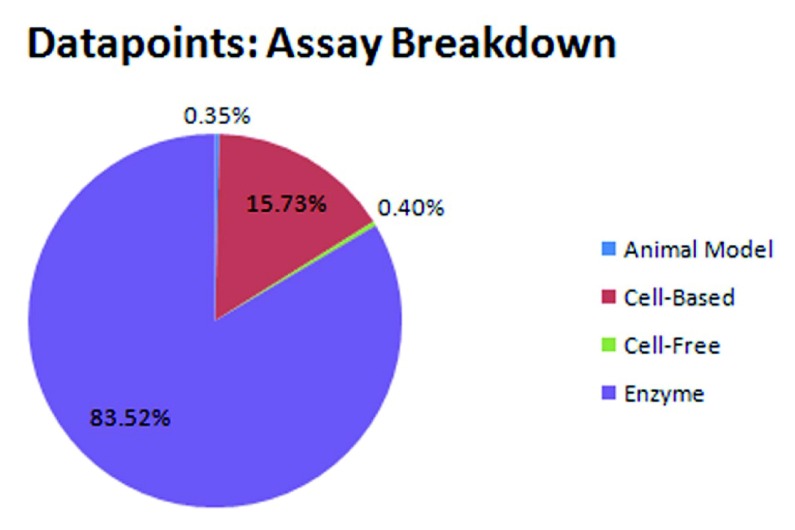
Data points share for each assay type.

**Figure 3.**
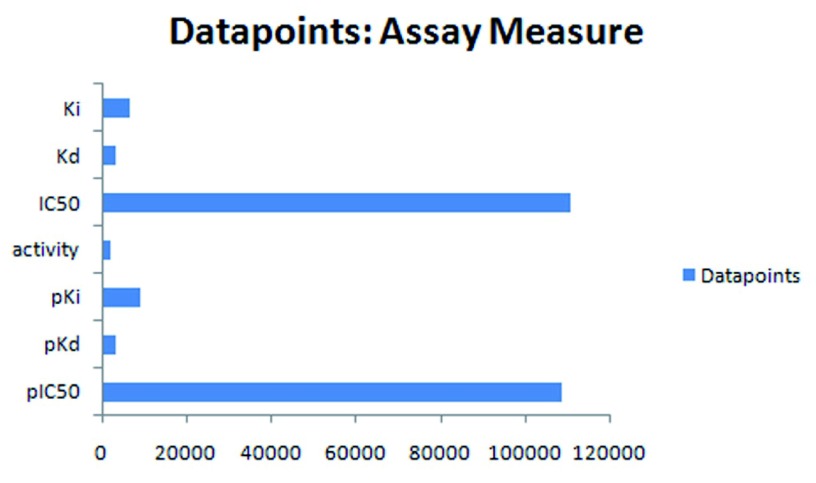
Data points in various assay measures.

The KKB validation sets have a maximum contribution from EGFR with nearly ~54K inhibitor molecules. This is followed by ~43K inhibitors for MAPK14; CDK2 and PIK3CA each have ~39K inhibitors. Figure 1 depicts data point distributions for each kinase in the attached subset. Moreover, 84% of the data are from biochemical enzyme based assay experiments, and 16% of the data from cell-based assays (in Figure 2). The datapoint measures include IC _50_, K _i_ and K _d_ ( Figure 3).

**Figure 4.**
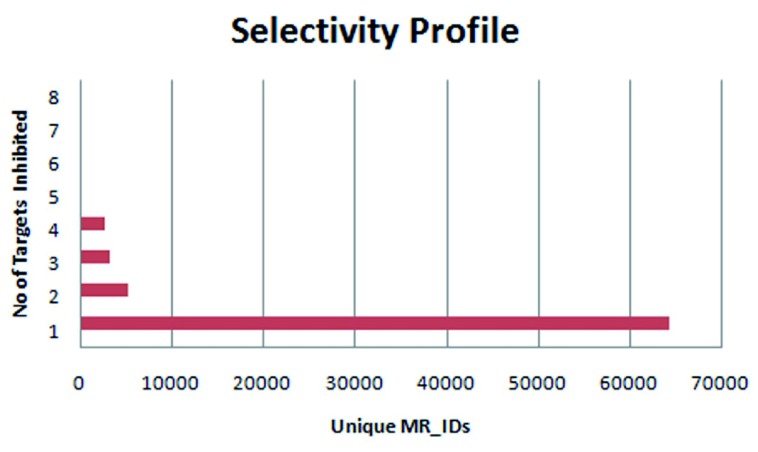
Selectivity profile for data points.

Analysis of ~76K unique molecules for selectivity against targets reveals that ~64K inhibit only one kinase of the eight kinases extracted ( Figure 4). Approximately 5K molecules show activity against two kinase targets, and ~3K molecules show activity against three kinases. A total of 79 molecules in the subset have some activity against all the eight kinase targets.

High quality, small molecule-activity for kinase research. Raw data behind the analyses described in the Data Note are included The file 'Datasets legends' contains descriptions for each dataset. Click here for additional data file. Copyright: © 2016 Sharma R et al. 2016 Data associated with the article are available under the terms of the Creative Commons Zero "No rights reserved" data waiver (CC0 1.0 Public domain dedication).

## Conclusions

The KKB is available in various formats such as SQL, SDF and IJC format ( Instant JChem) as quarterly updates. Two mobile apps, iKinase and iKinasePro ^25^, are also available for download which enable basic search access into KKB content, including kinase inhibitor structures, biological data and references/patents. Simple substructure and exact structure search access into the KKB is also available. We have extracted from the KKB ~258K structure activity data points and ~76K associated unique chemical structures across eight kinase targets and made these data freely available for download within this data note to foster algorithms development and validation worldwide.

## Data availability

The data referenced by this article are under copyright with the following copyright statement: Copyright: © 2016 Sharma R et al.

Data associated with the article are available under the terms of the Creative Commons Zero "No rights reserved" data waiver (CC0 1.0 Public domain dedication). 

F1000Research: Dataset 1. High quality, small molecule-activity for kinase research, 10.5256/f1000research.8950.d124591 ^36^
