# Supplementary material for: High quality, small molecule-activity datasets for kinase research
Source: F1000Res. 2016 Oct 26;5:Chem Inf Sci-1366. Originally published 2016 Jun 14. [Version 3] doi: 10.12688/f1000research.8950.3 (PMC4943296; doi:10.12688/f1000research.8950.3)
Supplement: Peer review history

# Peer review: Referee response for version 2

**DOI:** 10.5256/f1000research.9948.r15123

**Published:** 2016-07-21

**Authors:** Sorin Avram

**Affiliations:** 1 Department of Computational Chemistry, Institute of Chemistry Timisoara of the Romanian Academy (ICT) , Timișoara, Romania

I have read this submission. I believe that I have an appropriate level of expertise to confirm that it is of an acceptable scientific standard.

# Peer review: Referee response for version 1

**DOI:** 10.5256/f1000research.9629.r14358

**Published:** 2016-07-08

**Authors:** Sorin Avram

**Affiliations:** 1 Department of Computational Chemistry, Institute of Chemistry Timisoara of the Romanian Academy (ICT) , Timișoara, Romania

The paper describes Kinase Knowledgebase (KKB), i.e., a database containing structure-activity data on kinases. The current data note briefly presents the KKB Q1 2016 Release and the appended eight kinase data sets, which are made hereby publicly available. 

 Kinases are valuable targets for many diseases, especially cancers. The subject is of real scientific. In general, the amount of bioactivity data, coming from various sources (scientific literature, high-throughput screening results, patents etc), is heterogeneous and a proper curation and standardization of the data can provide reliable activity points. These data may be employed in many ways as described by the authors. In my opinion, the main applications for a database such as KKB would be to build predictors to search the chemical space for new kinase inhibitors, and further to optimize the selectivity of kinase inhibitors. Currently, ChEMBL’s ^1^ publicly available Kinase SARfari ( https://www.ebi.ac.uk/chembl/sarfari/kinasesarfari), provides a standard source for these tasks, covering about 532155 bioactivity data points i.e., version: 6.00- accessed June 20, 2016. This is less than one third of the 1.8 million KKB activity data point reported by the authors. In these circumstances, KKB might add valuable information for kinase research. Finally, the future analysis and employment of the eight data sets made freely available in the current note will provide a clearer view of the potential and versatility of KKB. 

 There would be two minor observations:

1. The methodology used to generate the data is described in the first paragraph in the section entitled “Kinase Knowledgebase (KKB)”. In order to be more accessible for the reader, this paragraph should be encompassed in a separate section named “Materials and methods”.
2. In Table 2 there are three columns with repeated headers. In order to remove any doubts, I would recommend the authors to clarify this issue.  

Otherwise, the data note is well written, kinases are indexed using the widely adopted Uniprot IDs and the references are updated.

 I recommend this data note for indexation and would like the authors to address the minor observations.

I have read this submission. I believe that I have an appropriate level of expertise to confirm that it is of an acceptable scientific standard.

## References

[1] BentoAPGaultonAHerseyABellisLJChambersJDaviesMKrügerFALightYMakLMcGlincheySNowotkaMPapadatosGSantosROveringtonJP: The ChEMBL bioactivity database: an update. *Nucleic Acids Res*.2014;42(Database issue) : 10.1093/nar/gkt1031 D1083-90 10.1093/nar/gkt1031 24214965PMC3965067

# Peer review: Referee response for version 1

**DOI:** 10.5256/f1000research.9629.r14833

**Published:** 2016-07-08

**Authors:** George Nicola

**Affiliations:** 1 Computational Biology, University of California at San Diego, San Diego, CA, USA

This article describes an overview of current kinase-related databases of significance, with particular focus on the contents of the Kinase Knowledgebase (KKB). The KKB has the largest repository of high-quality kinase activity data. Providing access to over ¼ million data points on several of the most important kinases allows for an exciting insight into the relevance of these validated drug targets and the diversity of compounds affecting them.  It is a promising trend that private companies are unlocking their proprietary data troves for the advancement of academic research. This is a nice Data Note that merits indexing in F1000Research.

 A few minor typographical corrections:

- Table 2: Three of the column names seem to be duplicated.
- Table 2: It is unclear what the grey vs white rows represent in ‘Kinase Classification’ and ‘Family’ columns. If only for readability, perhaps these should alternate.
- Section ‘Kinase inhibitor datasets’ at the end of the first paragraph: The word ‘respectively’ is not needed.
- Section ‘Kinase inhibitor datasets’ 4 ^th^ paragraph, ‘~54K inhibitors molecules’: ‘inhibitors’ does not need to be plural.
- Figures 1 & 2:I would use the word ‘Breakdown’ instead of ‘Breakup’.
- Figure 2: Are ‘Cell-Free’ and ‘Animal Model’ truly zero percent? If so, they should be excluded; if not, the fractional percent should be listed.
- ‘Conclusions’: ‘datanote’ should be two words, to be consistent with the F1000 article type.

I have read this submission. I believe that I have an appropriate level of expertise to confirm that it is of an acceptable scientific standard.
